# Study of the spatial strength distribution patterns and microstructure characteristics of ultra-fine tailings cemented paste backfill

**DOI:** 10.1038/s41598-024-83816-3

**Published:** 2025-01-02

**Authors:** Yuye Tan, Yiding Li, Xin Yu, Yuchao Deng, Zepu Song, Yunpeng Kou

**Affiliations:** 1 State Key Laboratory of High-Efficient Mining and Safety of Metal Mines of Ministry of Education, University of Science and Technology Beijing, Beijing, 100083 China; 2 School of Civil and Resources Engineering, University of Science and Technology Beijing, Beijing, 100083 China; 3 State Key Laboratory of Lithospheric and Environmental Coevolution, Institute of Geology and Geophysics, Chinese Academy of Sciences, Beijing, 100029 China; 4 Backfilling Engineering Laboratory Branch of Shandong Gold Mining Science and Technology Co., Ltd, Laizhou, 261441 Shandong China

**Keywords:** Ultra-fine tailings cemented paste backfill, Similar stope model, Spatial strength distribution, Electron microscopic scanning, Environmental sciences, Civil engineering

## Abstract

In this study, the spatial distribution of the mechanical strength of ultra-fine tailings cemented paste backfill (UCPB) in underground stopes was examined, and the micro-mechanism responsible for differences in spatial strength performance via changes in particle deposition was elucidated. To better understand this phenomenon, we constructed a similar backfilling stope model using the ultra-fine tailings of a gold mine. We manufactured specimens at different spatial locations and conducted a novel series of tests, including uniaxial compressive strength, shear strength, and conventional triaxial tests, to obtain the strength parameters in different spatial distributions. The strength parameters for the different UCPB locations in this model were revealed and examined along with the original pore and particle size distributions. Computer tomography (CT) and scanning electron microscopy (SEM) observations before and after the UCPB failed revealed that the particle deposition effect at different positions was the primary cause of the size effect. The strength distribution pattern demonstrated that the UCPB material has an apparent spatial effect. Consequently, considering the position relationship between the stope and filling mouth is crucial when designing the strength of adjacent mining rooms. This research may guide both backfill study and design.

## Introduction

Cemented paste backfill (CPB) technology has received increasing attention for effectively preventing surface subsidence and maintaining the stability of stope ground pressure^1,2^. An increasingly widely used technology in the filling mining method is referred to as paste filling. The waste rock and tailings generated in the production process are reused to increase their hydraulic filling capacity, and the concentrated filling slurry is transported to underground mining areas through drilling and pipelines^3–5^. At present, paste filling has become the primary choice and development direction of green mining in deep mines, which is highly important for controlling ground pressure and reduce the risk of goaf^6,7^.

With the depletion of many resources, the utilization efficiency of minerals has been improved, and environmental protection has been guaranteed. Through the application of advanced technology, crushed ore is finely ground to obtain ultra-fine tailings^8^. Some researchers define d_80_ ≤ 20µm as ultra-fine tailings^9^. Ultra-fine tailings have a relatively large specific surface area, which makes the physical and mechanical properties of the ultra-fine tailings cemented paste backfill (UCPB) different from those of CPB. Owing to the consideration of experimental conditions, in this work, only the spatial strength distribution of the UCPB is investigated. As a filling material, ultra-fine tailings have significant advantages, such as a large permeability coefficient, a short curing time and a fast water filtration speed^10,11^, which are conducive to the construction of tailless mines with ecological resources. However, to date, there have been few studies on the spatial strength distribution of UCPB.

Unconfined compressive strength (UCS), direct shear strength, and triaxial compression tests are three efficient methods for evaluating the mechanical properties of CPB. In existing studies, scholars have constructed damage evolution models, damage constitutive models and strength criteria^12–14^ for CPB. The effects of different mix ratios^13^, different particle size distributions^15^, various flocculants^16,17^, and different water saturations^18^ on the internal pore structure and mechanical properties of CPB have been studied in detail. The spatial distribution structure of key materials in CPB also affects the overall performance of the backfill^19,20^. Lu^21^ et al. revealed the structural characteristics and distribution patterns of strength in backfill materials to understand their deformation behavior and failure modes under different confinement pressures for heterogeneous dense specimens. Lei^22^ et al. reported that the spatial distribution of defects in concrete may conform to either the Poisson assumption or the law of uniform distribution, whereas the statistical characteristics of concrete strength do not consistently align with Weibull statistics. Massimo^23^ et al. introduced the main global guidelines for the statistical analysis of concrete core test data and investigated the strength characteristics of the CPB in a stope, which, in a similar stope model, can be attributed to sediment transport as well as macro and micro characteristics^24^. Therefore, it can be inferred that CPB has four different intensity regions^25–28^.

The existing research and analysis methods have mainly focussed on the local mechanical strength of backfill materials and lack of systematic research on the spatial strength distribution of large-scale backfill materials. At construction sites, ultra-fine tailings consolidated backfill^29–32^ is heterogeneous, and its strength is affected by many factors, such as particle size distribution^33–35^, temperature field^36–38^, and spatial location. Therefore, to compensate for the lack of research on the spatial strength distribution of large-structure backfill, in this paper, a simulated quarry test is designed on the basis of the actual filling quarry. It consists of measurements of the mechanical strength characteristics of the fill at different locations. This study elucidates the spatial distribution of backfill strength^39^. Computed tomography (CT) and scanning electron microscopy (SEM) are used to analyze the spatial evolution of the internal strength of the backfill from a microscopic perspective.

## Materials and methods

### Materials

The present study was conducted by using the ultra-fine tailings exclusively sourced from a gold mine situated in Shandong Province, China. Here, the scaled simulation model (SSM) included tailings, cement backfilling materials (material C), and water. Material C was independently manufactured by the laboratory of Shandong. For the design, tap water was used to prepare backfill to improve the consistency between laboratory tests and the field environment.

**Fig. 1:**
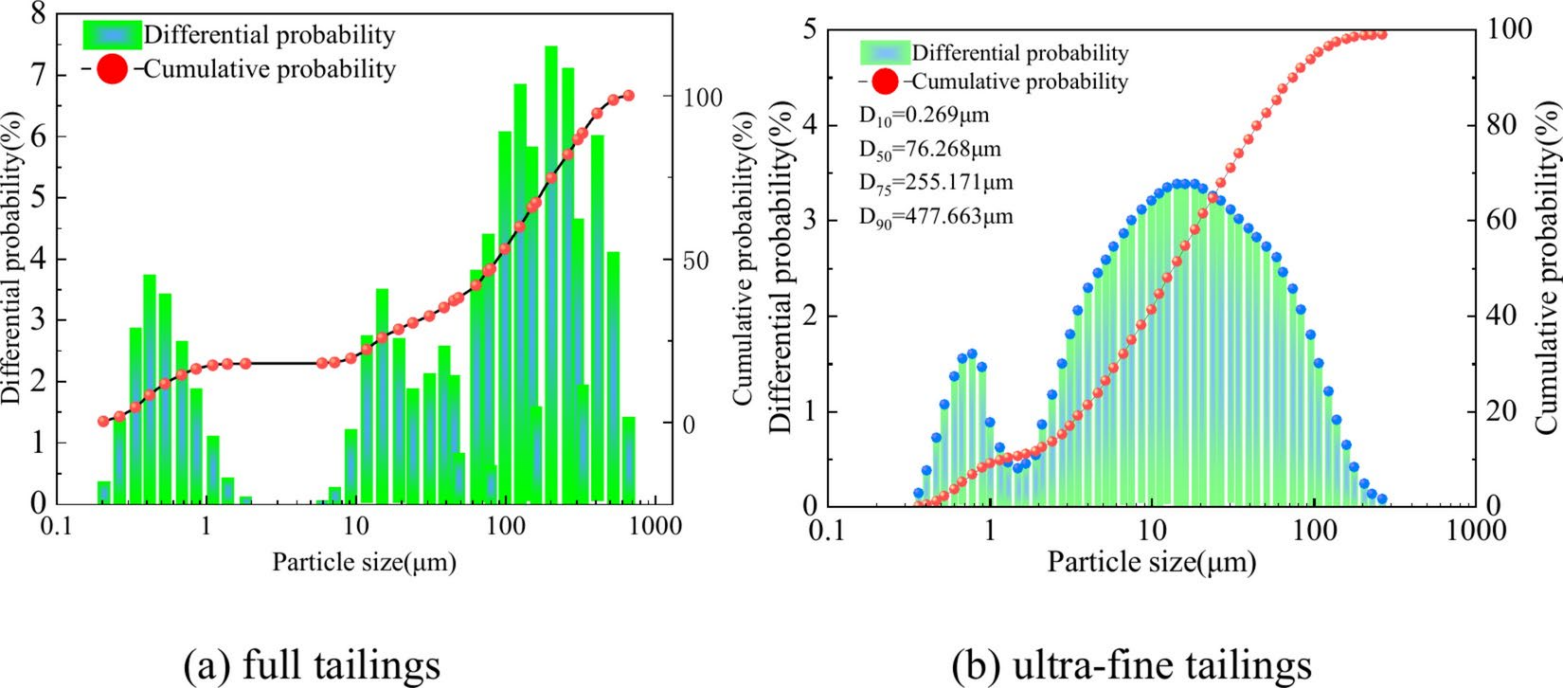
Tailings particle size analysis.

**Fig. 2:**
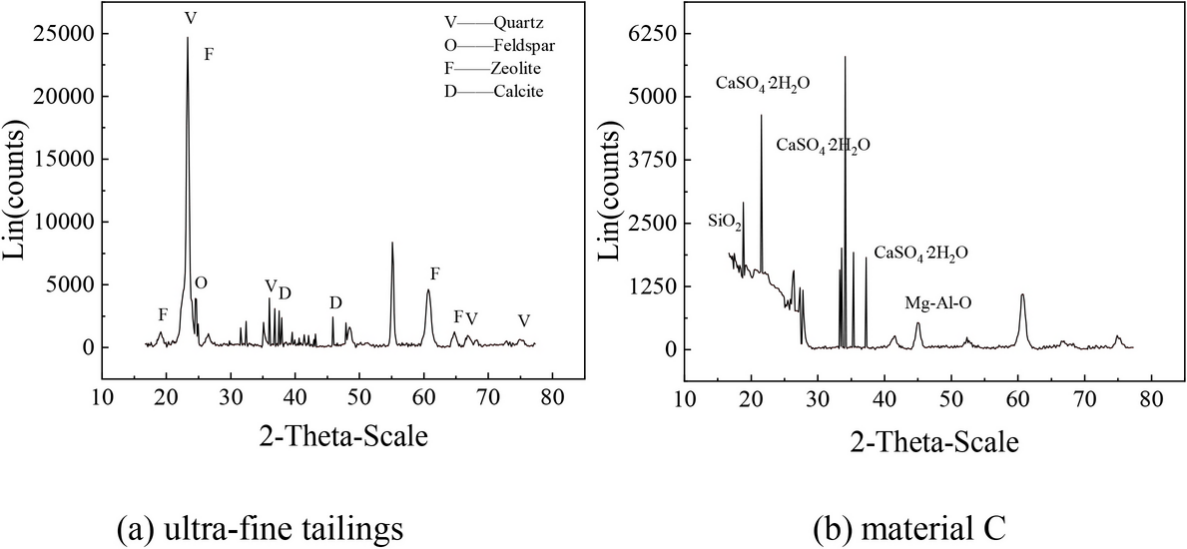
XRD phase analysis.

A Malvern laser particle size analyzer was used to determine the particle size distributions of full tailings and ultra-fine tailings (Fig. 1). The ultra-fine tailings had an actual density of 2.69 g/cm^3^ and a more uniform particle size distribution, with a range of 0.357 ~ 272 μm and d50 = 25.29 µm. A comparison of the full tailings with the ultra-fine tailings revealed that the particle size distribution of the fine tailings was more uniform. The non-uniformity coefficient Cu and curvature coefficient Cc of the ultra-fine tailings are 16.611 and 1.561, respectively. Their particle size is smaller than that of ordinary Portland cement particles, so it is difficult to form a filling body with a stable structure and good performance, which may be deemed necessary in order to mitigate the adverse effects caused by the backfill material. The X-ray fluorescence (XRF) results revealed (Table 1) that the predominant chemical composition present in tailings is SiO_2_, MgO Fe_2_O_3_, etc. Figure 2 shows the X-ray diffraction (XRD) results, which indicate that the primary crystalline phases in tailings are quartz, amphibole, mica, and endenite. This shows that the ultra-fine tailings have active properties and can generate a large amount of calcium silicate hydrate (C-S–H) gel in the hydration reaction. The spectral analysis in Fig. 2 revealed that the backfilling material C contains a significant number of active oxides, including calcium oxide, which can form active hydration products such as tricalcium silicate (C_3_S), tricalcium aluminate (C_3_A), and gypsum. The ratios of various materials constituting Material C are presented in Table 2. These results indicate that the backfilling material C has excellent cementitious properties.

**Table 1:** XRF chemical analysis of ultra-fine tailings.

Ingredients	SiO_2_	Al_2_O_3_	CaO	Fe_2_O_3_	K_2_O	MgO	Na_2_O	TiO_2_
Content %	61.754	12.68	9.1819	5.952	4.436	3.304	1.243	0.6499

**Table 2:** XRF chemical composition analysis of the backfilling material C.

Ingredients	CaO	SiO_2_	Al_2_O_3_	MgO	SO_3_	Fe_2_O_3_	TiO_2_
Content %	44.57	29.95	10.09	6.639	5.295	1.251	0.722

### Methods

#### Scaled simulation model (SSM) establishment

**Fig. 3:**
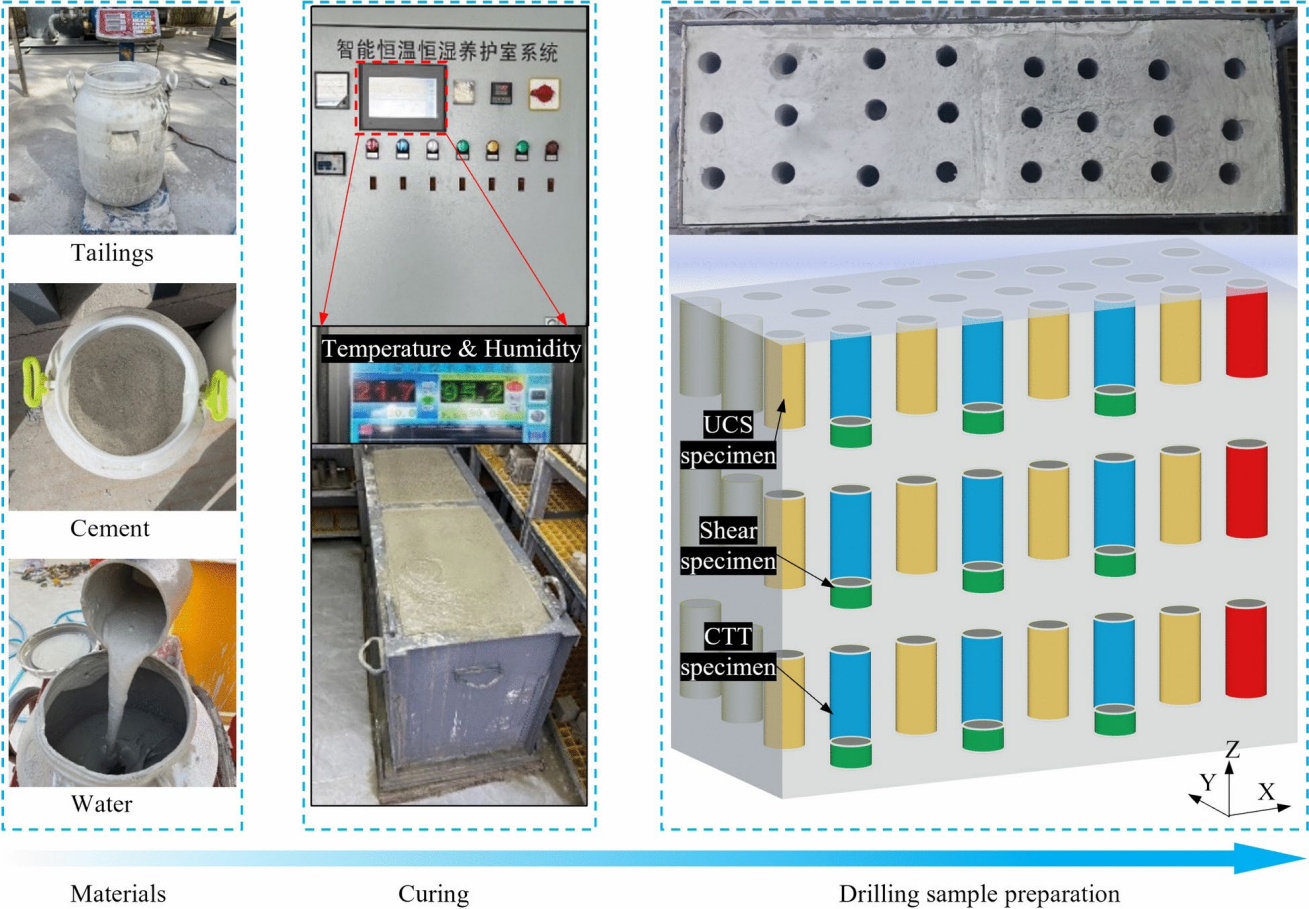
Similar stope model.

Via the similarity principle, a similar backfilling stope model was established according to the proportion of 1:30, taking a stope of a gold mine as the prototype. In a similar model, the mechanical properties of the UCPB were tested, and the microstructure was observed to study the spatial strength distribution of the UCPB. The dimensions of the stope were adjusted proportionally 1600 mm × 450 mm × 500 mm (length × width × height). Employing the Reynolds similarity principle as a guide, the backfilling flow rate was calibrated at 60 m^3^/h. The structure of the solidification device in the physical similarity model is illustrated in Fig. 3. Using a combination of steel plates and acrylic materials, the SSM was engineered to facilitate slurry transport via gravity-induced self-flow, mirroring the dynamics of a mine backfilling procedure. To ensure optimal functionality, the conduit connecting the blending device and the lower section of the funnel was designed with a 5 mm diameter.

Traditionally, the most commonly employed drainage methods within backfill stopes involve the utilization of central artificial drainage wells and peripheral drainage pipes positioned along the stope’s boundary. To replicate these conditions, a water filter screen composed of a steel mesh wrapped in fabric was meticulously placed at the extremity of the model. This arrangement closely emulates the boundary drainage mechanism inherent to the SSM, thereby increasing the comprehensiveness and accuracy of the system’s representation.

#### Specimen preparation

The ratio of the mixture for the experiments was kept the same as that in situ, namely, a mass fraction of 64% and a cement/tailings ratio of 1:4. The tailings, material C, and water were blended thoroughly after being measured, and the values were calculated. Continuous backfill technology has been developed in the SMM to accommodate the unique space environment of the mining areas. Thus, after being stirred for about approximately 5 min, the paste should be continuously and steadily injected into the SSM through pipes, with valves on the pipeline connected to the mixer controlling the process, as shown in Fig. 3. Note that the mixer remains operational for a uniform slurry consistency. To match the on-site environment, in this experiment, the temperature was controlled at approximately 20℃, and the humidity was maintained at approximately 80% by an intelligent constant temperature and humidity maintenance system. The maintenance system maintained the test environment by periodically delivering water vapor. The humidity was 90%. Mechanical tests and microstructural analysis were conducted on the UCPB specimens at 28 days.

#### Specimen coring design

**Fig.4:**
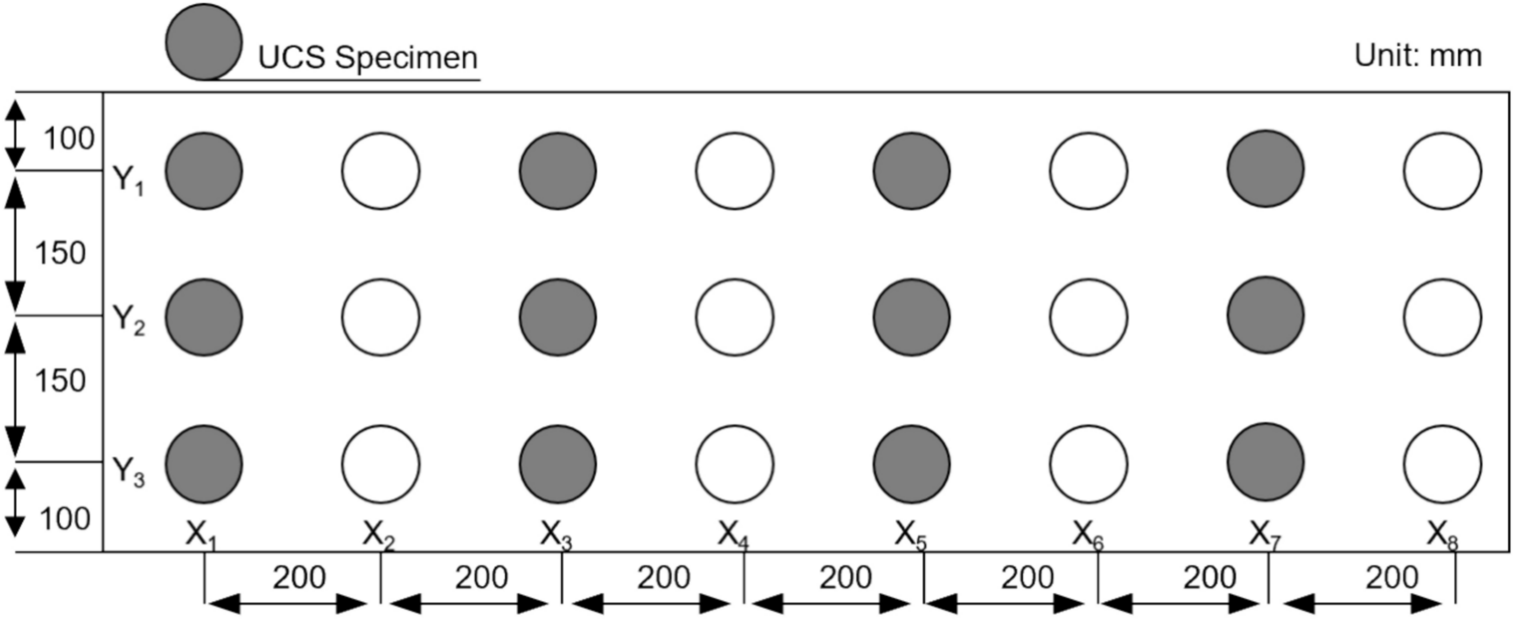
Test piece and equipment.

**Fig. 5:**
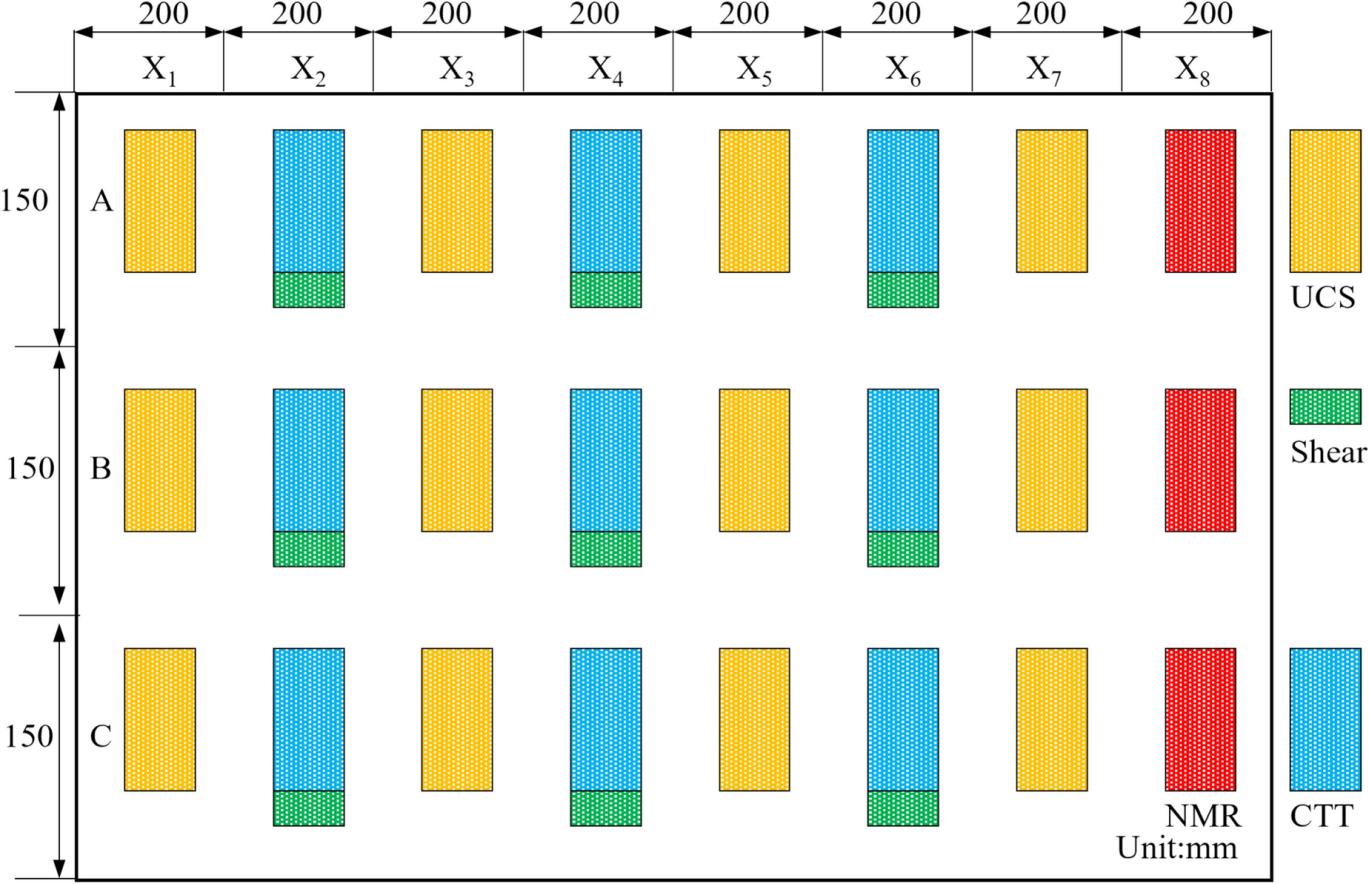
Sampling locations of the CTT specimens and shear specimens.

Evaluating the comprehensive strength of the UCPB at various spatial positions requires the introduction of a nomenclature on the basis of their spatial distribution patterns (Fig. 4). The specimen spacing was 200 mm in the X direction and 150 mm in the Y direction. We employed a method that involves processing equally spaced specimens (Fig. 5). In this approach, we utilized the principle of parity allocation to select specimens for various mechanical tests. Because the specimens exhibit distinct failure modes under various stress states, there is only one chance for testing and determination. Interval sampling ensures the comprehensive acquisition of the specimen’s failure modes at the same location.

As shown in Table 3 along the X axis, 36 even-numbered specimens are designated for uniaxial compressive strength tests (UCS), whereas odd-numbered specimens are designated for 27 conventional triaxial tests (CTT) and 45 direct shear tests. The sample size for the uniaxial compression test and triaxial compression test was 100 mm × *Φ*50 mm, and the sample size for the shear test was 25 mm × *Φ*50 mm. The production and experimental measurements of the specimens were conducted in accordance with the following specifications: Code for rock tests in water and hydropower projects (SL/T2642020) and Standard for test methods of engineering rock mass (GB/T 50,266–2013).

**Table 3:** Specimen parameters.

Tests	Diameter (mm)	Height (mm)	Confinement (MPa)	Total amount
UCS	50	100	0	36
CTT	50	100	0.3, 0.5, 0.7	27
Direct shear	50	25	0	45

**Fig. 6:**
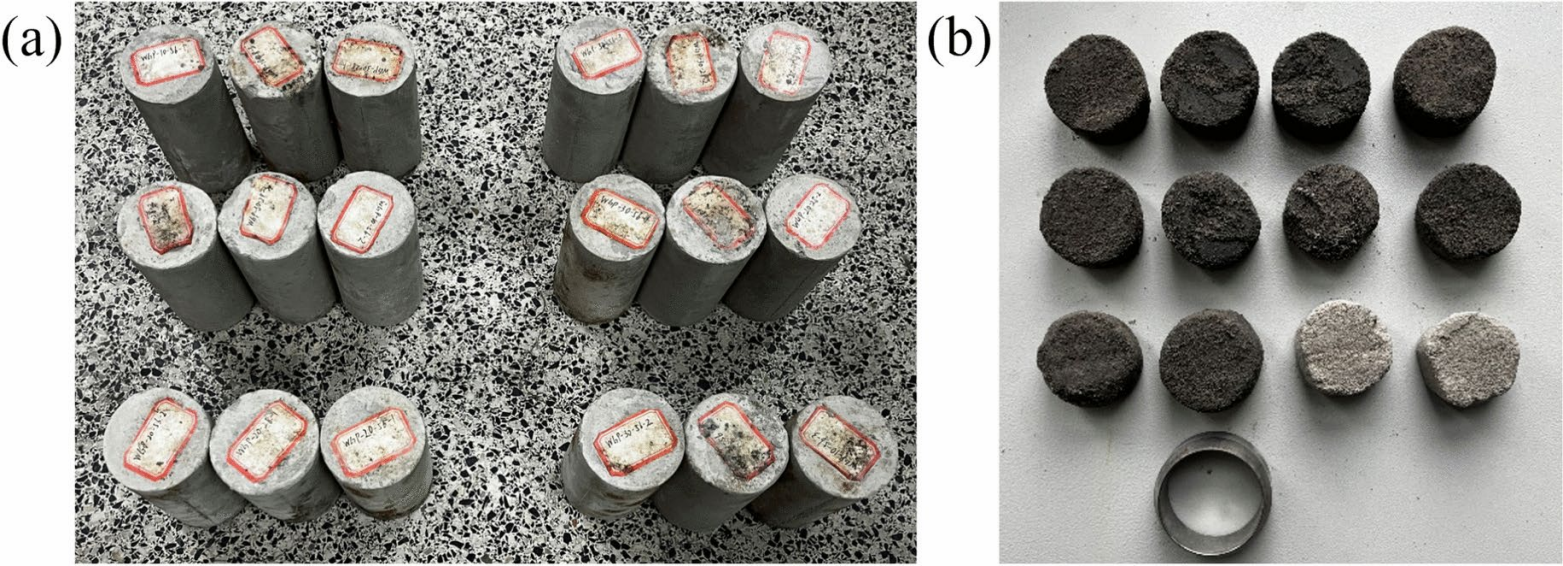
Test specimens of (**a**) UCS and CTT and (**b**) direct shear.

As an illustration, the label “X_3_Y_8_A” signifies that the specimen is situated in the top region of row 3 and column 8. The specimens were cored, cut, and polished to various sizes, as depicted in Fig. 6.

### Experimental equipment

#### Mechanical experiments

**Fig. 7:**
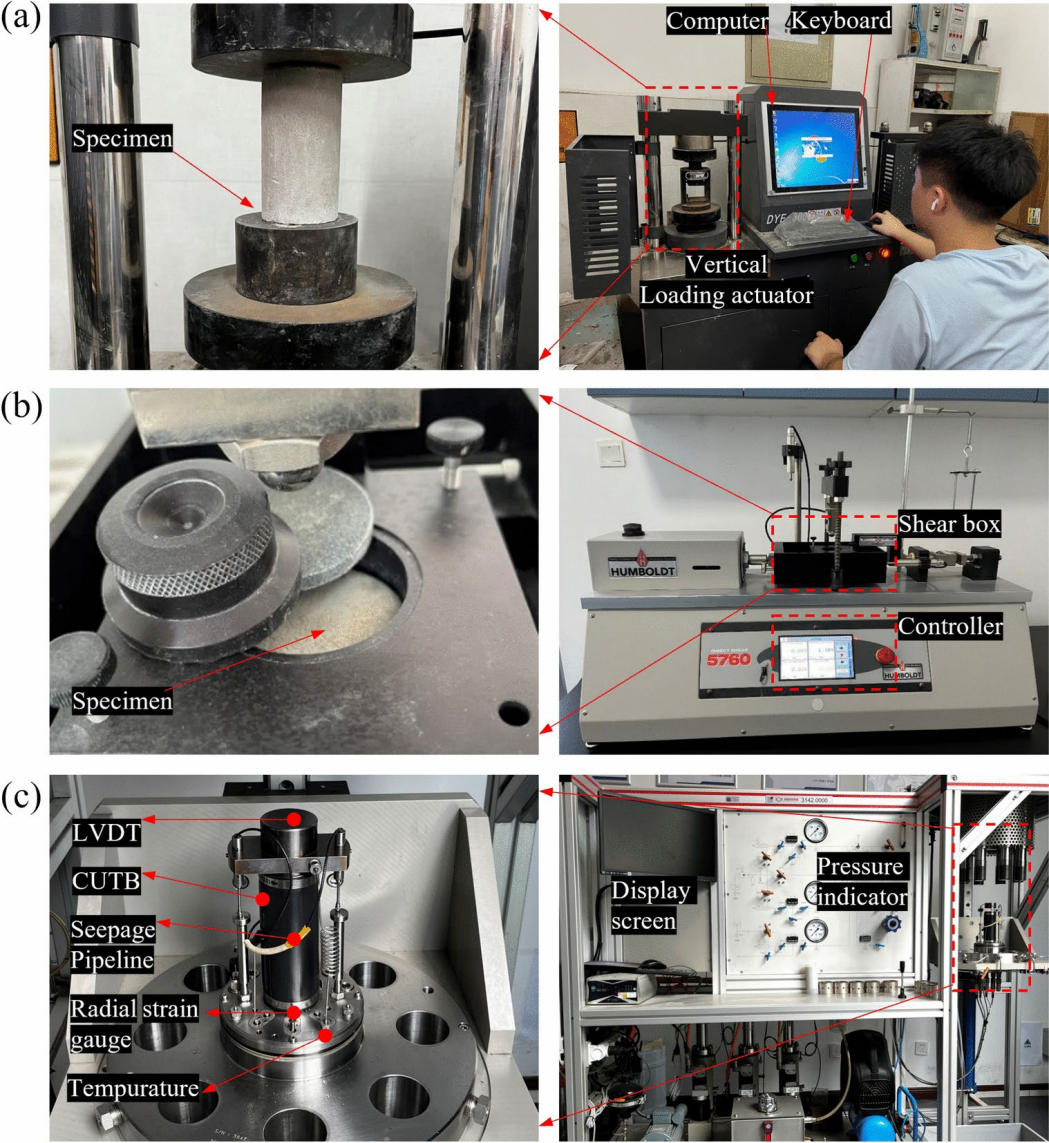
Experimental equipment for (**a**) UCS, (**b**) direct shear, and (**c**) CTT.

An electronic universal testing machine equipped with the a YAW-200C rock mechanics servo-controlled testing apparatus (as shown in Fig. 7(a)) was employed to ascertain the UCS. Given the comparatively low UCS of the UCPB (approximately one-tenth that of conventional concrete), a loading rate of 1 mm/min was chosen. The final strength was determined as the average UCS value from three parallel specimens.

The HUMBOLDT-5760 direct shear apparatus (as depicted in Fig. 7(b)) uses a hydraulic pump to generate pressure. This pressure enables the shearing of specimens through the relative displacement of two halves of shear boxes—one fixed and one movable. During the shearing process, the upper shear box remains stationary while loading, whereas the lower box is propelled by a motor at a consistent velocity of 1 mm per minute until a jawless residual shear strength (residual strength) is achieved. The shear box is linked to a digital control system comprising a computer, software, and a data acquisition system. To measure the shear strength parameters (internal friction angle and cohesion) of the UCPB, three normal stresses of 50 kPa, 110 kPa, and 170 kPa are applied.

Compressive triaxial compression tests were conducted on specimens via a 600 kN loading machine that applied strain control while maintaining a confining pressure of 2 MPa. The triaxial compression equipment consists of a pressure chamber, a pore pressure measurement system, an axial load application system, a confining pressure system, and a volume change monitoring system. The test specimen was placed between porous stone caps, and then a latex membrane was installed around the specimen. The specimens were tested throughout the testing procedure at a deformation rate of 0.05 kN/min, with the computer automatically capturing and storing the associated data. The confining pressure was first applied to the designed values, and then axial strain loading was applied until the CPB-rock specimen failed in accordance with ASTM D4767-11[38]. The confining pressures were set to 0.3 MPa, 0.5 MPa, and 0.7 MPa. Figure 7(c) shows the three-axis compression testing apparatus employed in conjunction with the experimental control system for the UCPB specimens.

#### Microstructure experiments

The presence of water complicates the establishment of a stress state in tailing-based backfill. The pore structure of a material was determined by magnetizing the water molecules within its pores, enabling the acquisition of the nuclear magnetic resonance (NMR) T_2_ spectrum. The Meso MR23-060H-I analysis system serves as the experimental equipment employed in this study. The time calculation commenced upon the mixing of aggregate and water. Following a brief pause of 5 min, the initial assessment of relaxation time T_2_ was conducted, with data being collected at designated intervals (i.e., 6 h, 12 h, and 24 h) throughout the curing process.

**Fig. 8:**
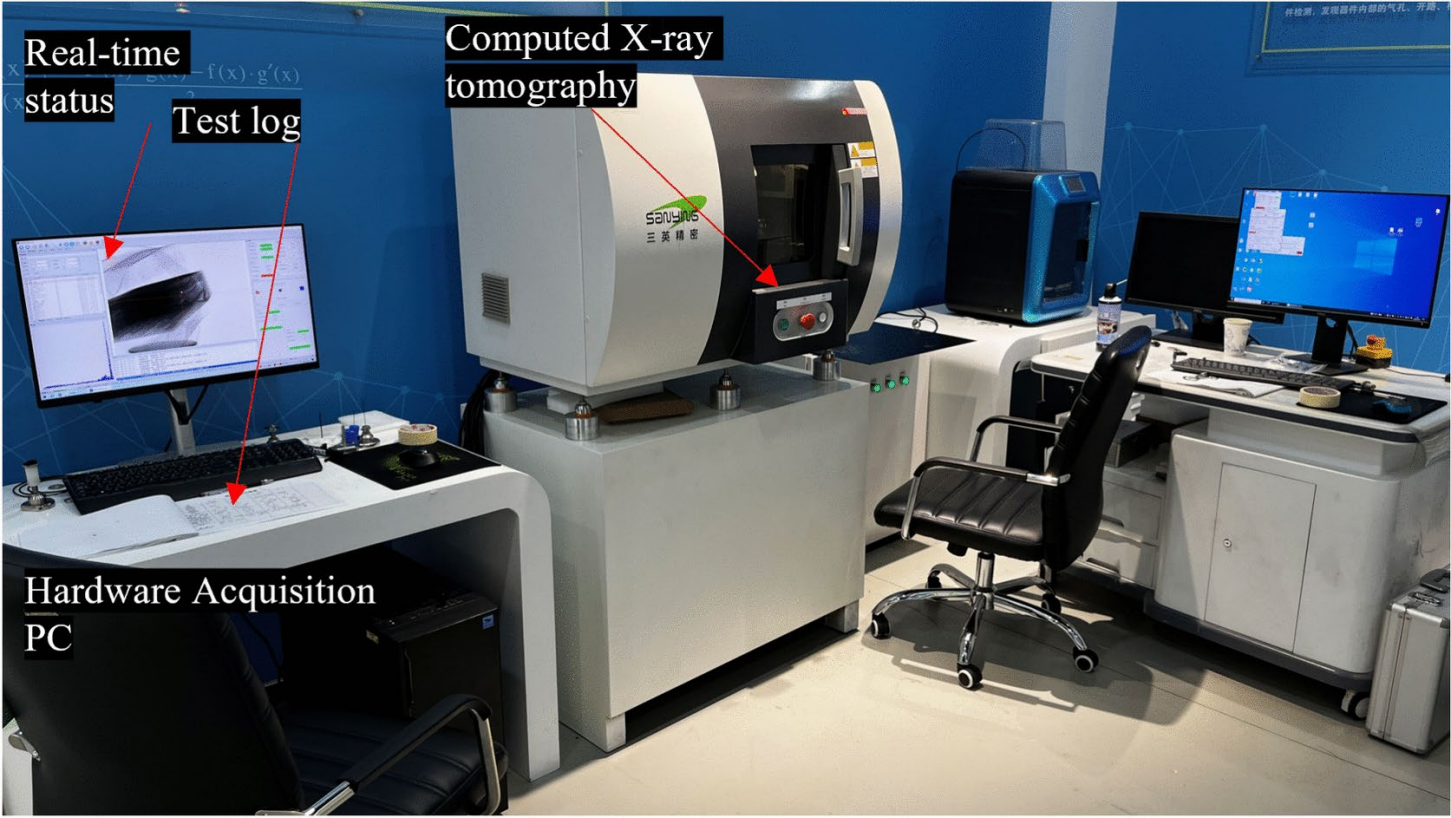
CT equipment.

X-ray computed tomography (CT) is a non-invasive technique used to visualize internal structures within cementitious materials such as UCPB, enabling the acquisition of digital information regarding their three-dimensional (3D) configurations and characteristics. Reconstructing 3D images from internal structural attributes requires determining the CT resolution on the basis of various factors, including the dimensions of the amplified photon source, the distances between the source and specimen, and the distances between the specimen and the detector, which are ultimately considered (Fig. 8). Owing to significant variations in X-ray absorptivity among materials with different densities, a detector system equipped with a flat panel is employed to obtain comprehensive scan results. The system comprises an X-ray source, head, and detector subsystems featuring a flat panel, a scan device/control subsystem, and a rebuilding subsystem. The essential parameters of a CT scan include a aspatial resolution of 3 lp/mm, an energy value of 6 MeV, and the ability to detect steel thicknesses of up to 196 mm. A total of 2700 slices were obtained along the parallel loading direction, with 8-bit grayscale images, a resolution of 2000 for each specimen, and a pixel interval of 36.6 µm.

The fracture surface and microstructure properties of a break in the UCPB were analyzed via SEM after the UCS test along the fracture surface, and a the relatively flat fracture surface was selected. Before this experiment, a layer of copper foil was wrapped on the outside of specimens, then carbon was sprayed, and the specimens were placed in a vacuum room for 30 min. The experimental equipment used was a Carl Zeiss tungsten filament SEM Evo 18. The images were acquired at a resolution of 20 µm, using an accelerating voltage of 20 kV, an amplification factor of 2000, and a primary energy level of 20 keV. SEM observations can provide deep insights into a broken section and its particle distribution.

## Results and analysis

### Spatial distribution of the UCS

**Fig. 9:**
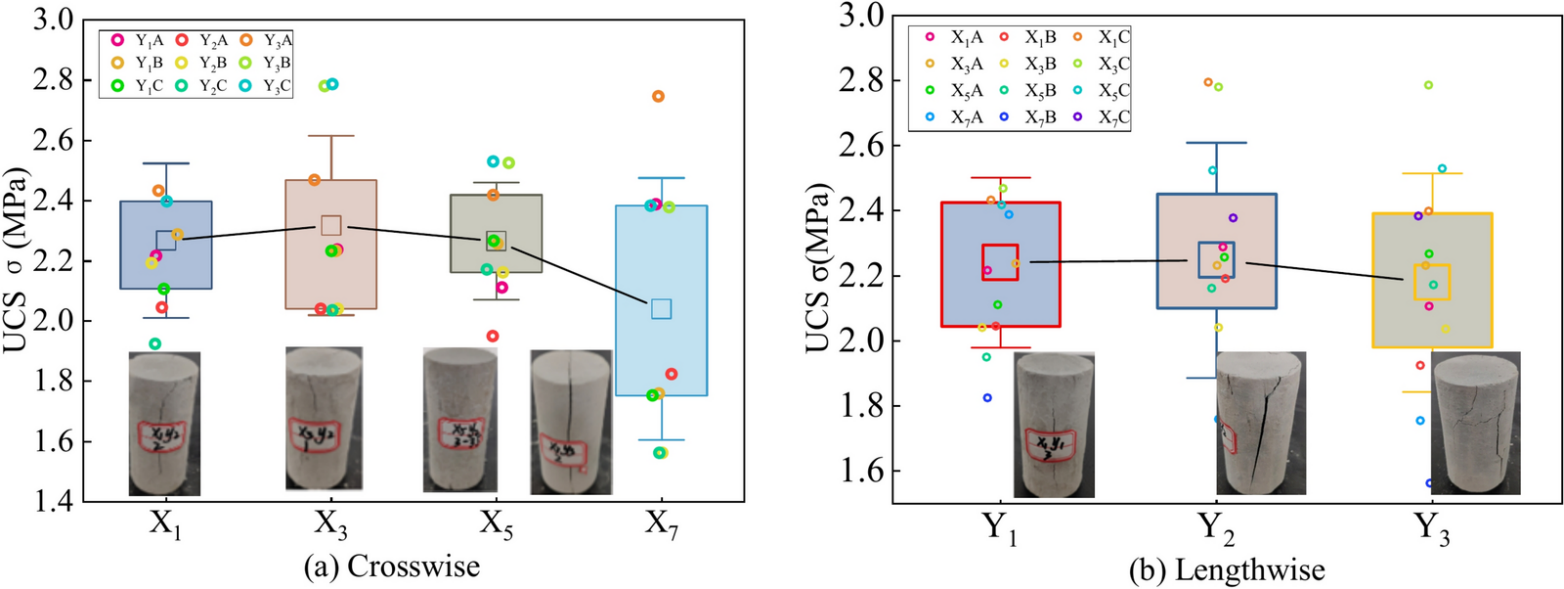
UCS test results of the UCPB: (**a**) crosswise distribution and (**b**) lengthwise distribution.

The strength distribution of the UCPB exhibits distinct regular characteristics and nonuniformities. Figure 9(a) illustrates the influence of plane orientation on strength, with an average UCS of approximately 2.22 MPa. According to the statistical results, there was a noticeable trend of increasing UCS values from the periphery to the center. In terms of descriptive statistics presented through frequency charts, the average strength initially increased from 2.26 MPa to 2.31 MPa, followed by a decrease from 2.31 MPa to 2.22 MPa as the location progressed from the beginning to the end. Additionally, the UCS range distribution was observed to be discrete across horizontal distances. Furthermore, the strength distribution clearly widened with increasing spacing. This can be influenced by several factors, including mineralogical composition, dimensions, morphology, and spatial distribution of mineral grains^39^. The error line span in Fig. 9 gradually increases as the position moves, indicating a more comprehensive fragmentation of the UCPB specimens and a gradual convergence towards an unstable distribution.

The effect of longitudinal distance on the UCS is illustrated in Fig. 9(b). On the basis of the data distribution, as the lateral extension distance increases from 0 mm(Y_1_) to 500 mm(Y_3_), the median UCS expands from 2.17 MPa to 2.24 MPa. In terms of the longitudinal direction, both the strength and range show an increasing trend. However, when considering the overall perspective, this spatial difference is not as pronounced as it is in the horizontal direction. Notably, there is a significant range of strengths in the longitudinal direction at 0.07 MPa, which accounts for approximately 3% of the average strength and demonstrates a stable distribution pattern. Consequently, UCPB exhibits uniform strength characteristics along its longitudinal axis with consistent material flow at interfaces.

**Fig. 10:**
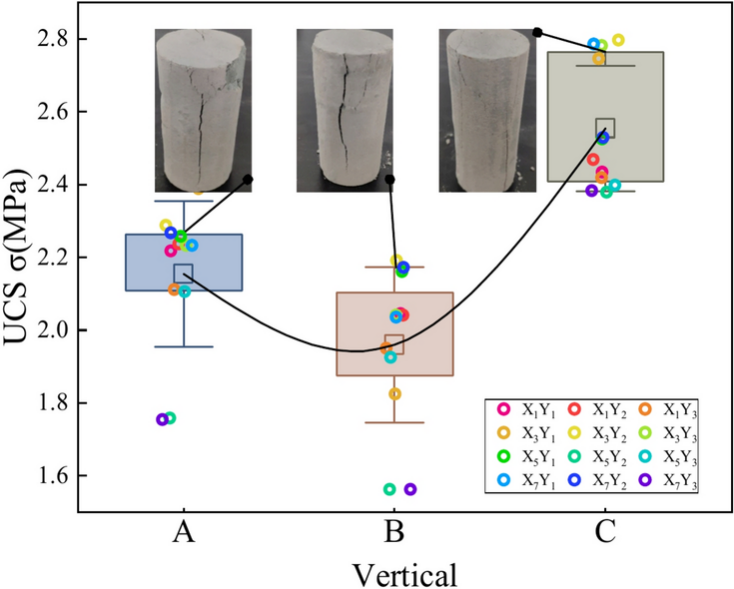
Spatial characteristics of the vertical strength.

Figure 10 illustrates the evolution of the uniaxial compression damage characteristics and strength of borehole X_5_Y_2_. The strength of the UCPB changes from a decreasing trend to an increasing trend from position A to C, showing an overall increasing trend.

### Spatial distribution of the CTT

**Fig. 11:**
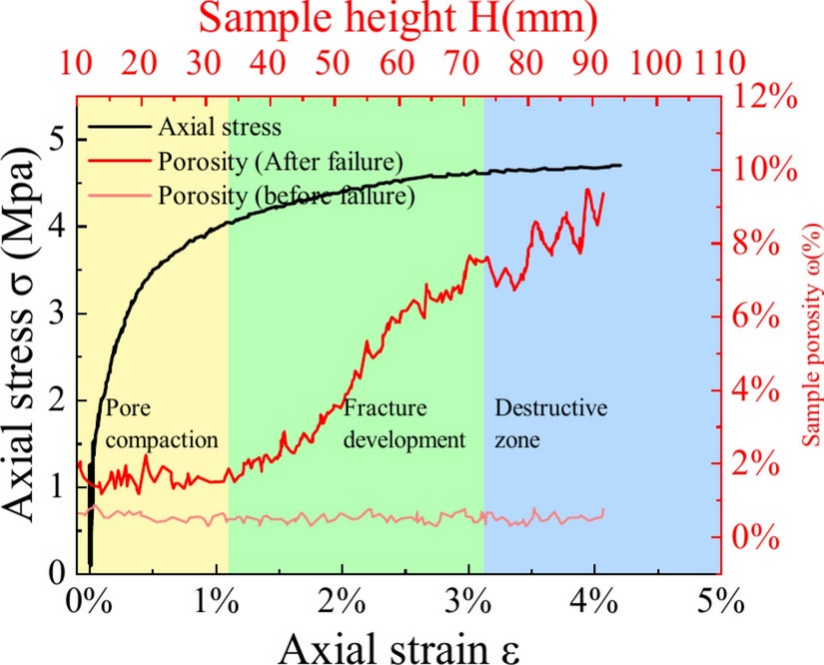
X_6_Y_3_ Stress‒strain curve in CTT.

**Fig. 12:**
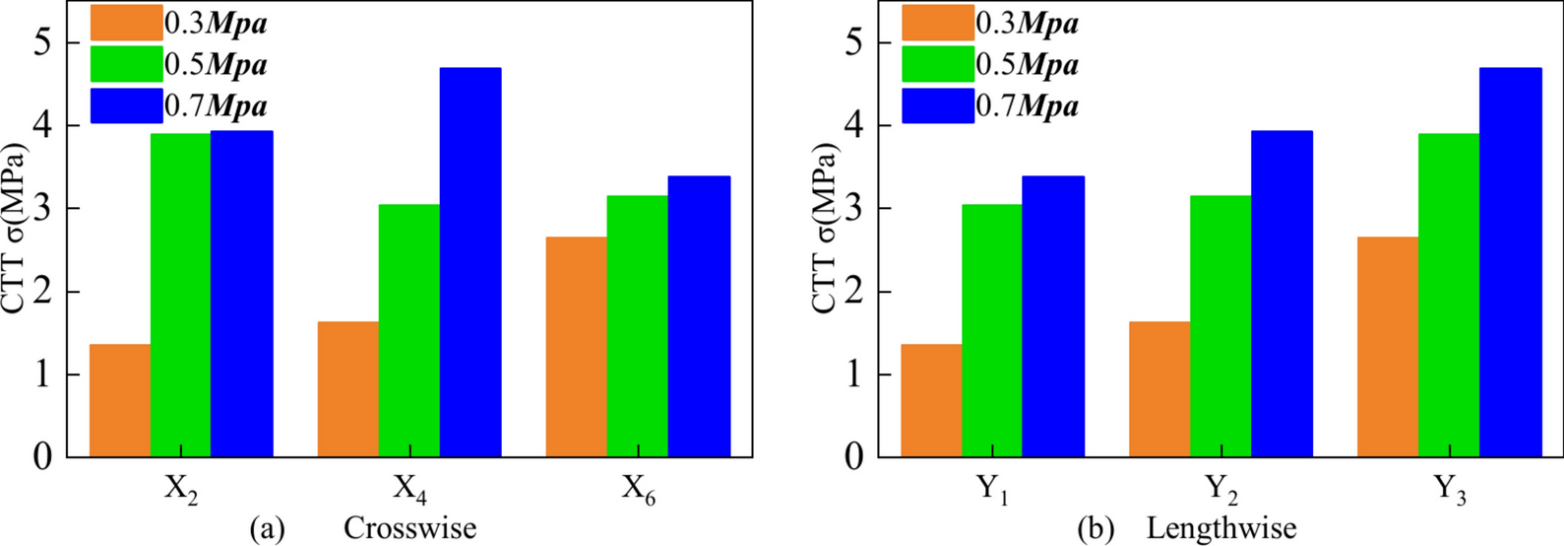
CTT test results of the UCPB: (**a**) crosswise distribution and (**b**) lengthwise distribution.

**Fig. 13:**
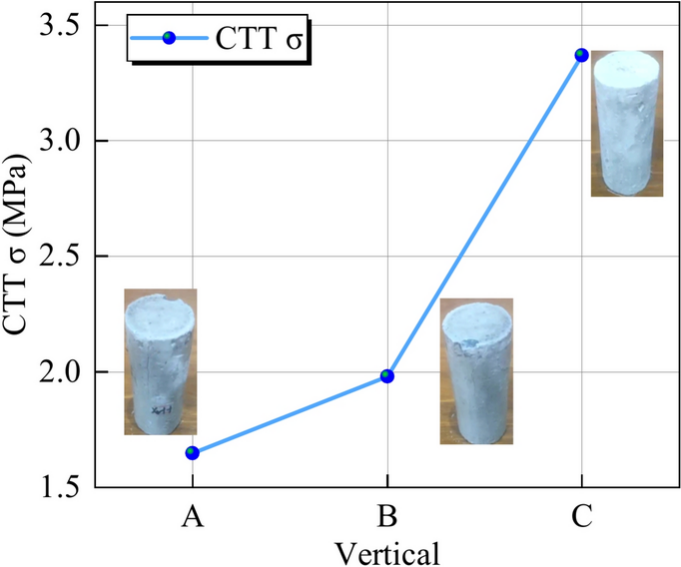
Vertical distribution and failure mode of the CTT.

The test parameters and results are presented in Table 4, and the stress‒strain curves of the specimens are shown in Fig. 11. Specimen X_6_Y_3_ is taken as an example to illustrate the general fracture characteristics of the CTT test. Figure 12 shows the spatial distribution of triaxial strength under different circumferential pressures. The analysis results show that the triaxial strength is sensitive to the longitudinal direction, and that the strength tends to increase with increasing distance. In the crosswise distribution, the strength is greater at the middle position. In the same spatial area, the compressive strength of the UCPB increases with increasing surrounding pressure. The effect of the strength of the UCPB in the spatial distribution lies primarily in the different stress states. This finding confirmed that the controlling factor in the buckling behavior of the UCPB is the lateral constraint of the rock. According to the spatial stress distribution of the actual backfilling quarry, the compressive state of the backfilling body is reasonably paraphrased. Moreover, as depicted in Fig. 13, a discernible trend emerges wherein the vertical intensity distribution escalates with increasing depth.

**Table 4:** Mechanical parameters of the UCPB.

NO	Label	Triaxial strength (MPa)	Modulus of elasticity (GPa)	Poisson’s ratio
1	X_2_Y_1_	1.35	0.18	0.023
2	X_2_Y_2_	3.92	0.21	0.023
3	X_2_Y_3_	3.89	0.20	0.026
4	X_4_Y_1_	3.04	0.17	0.018
5	X_4_Y_2_	1.62	0.22	0.026
6	X_4_Y_3_	4.69	0.20	0.025
7	X_6_Y_1_	3.38	0.19	0.021
8	X_6_Y_2_	3.14	0.19	0.022
9	X_6_Y_3_	2.65	0.19	0.026

### Direct shear test

**Fig. 14:**
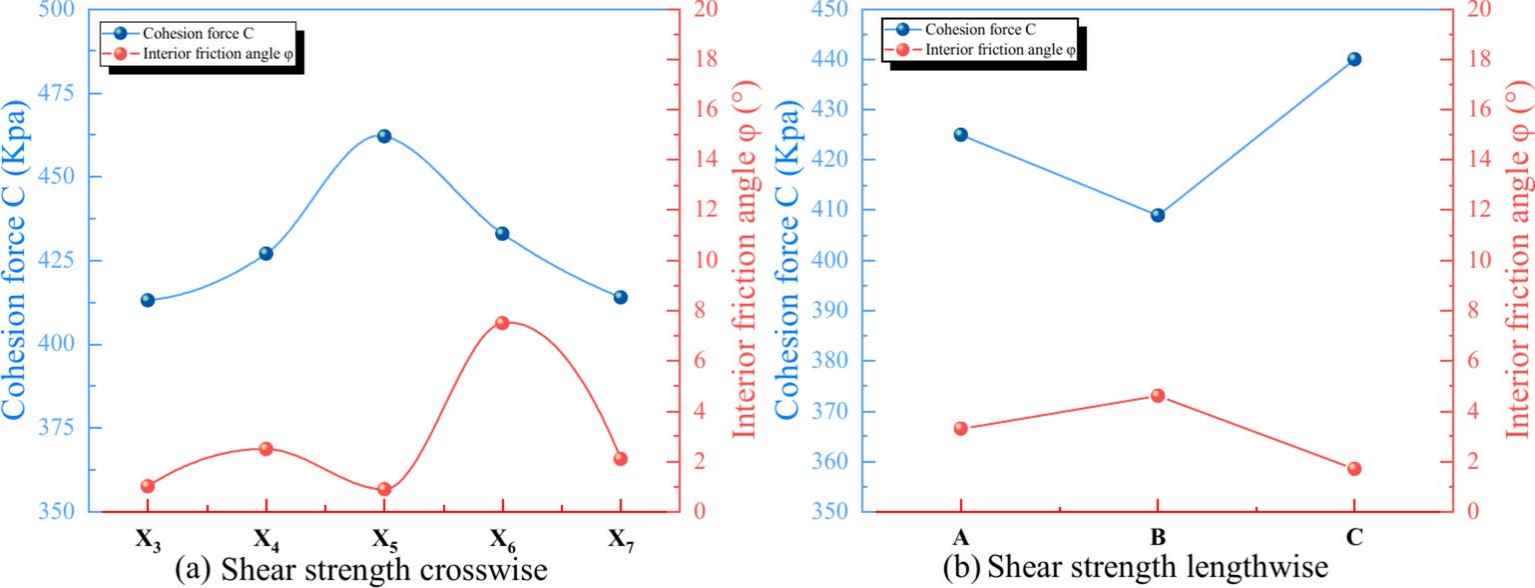
Shear spatial strength evolution of the UCPB: (**a**) crosswise and (**b**) lengthwise.

The spatial distribution of the shear strength of the UCPB is illustrated in Fig. 14. The transverse variation trend of the internal friction angle aligns with that of the cohesion, with both exhibiting a gradual increase, followed by a decrease. The cohesion attains its highest value at the midpoint of the UCPB while being relatively lower on the flanks. Similarly, the friction angle exhibits an ascending and then descending pattern, with its peak value situated at the central part of the backfilling body, tapering off towards the sides. This analysis reveals that within the entire matrix, the positions displaying the maximum cohesion and internal friction angle are proximate to the middle of the UCPB.

Interestingly, a contrary strength transformation is observed longitudinally. Moreover, for a given cross-section, the longitudinal position can be linked to a rate of change in cohesion fluctuation. For example, the cohesion at positions Y_1_ (0.42 MPa) and Y_3_ (0.44 MPa) increases by of 3.76% and 7.05%, respectively, in relation to the 28-day UCPB at position Y_2_ (0.89 MPa). Figure 14(b) also portrays the cohesion and internal friction angle results of the lengthwise UCPB. This visualization indicates that the UCPB at position Y_2_ experiences a trough, which correlates with a corresponding cohesion value of 0.41 MPa. However, this conclusion is based on only three specimens, and additional experiments are needed for validation in the future. Notably, the findings underscore a consistent lateral distribution trend around the flow direction.

**Fig. 15:**
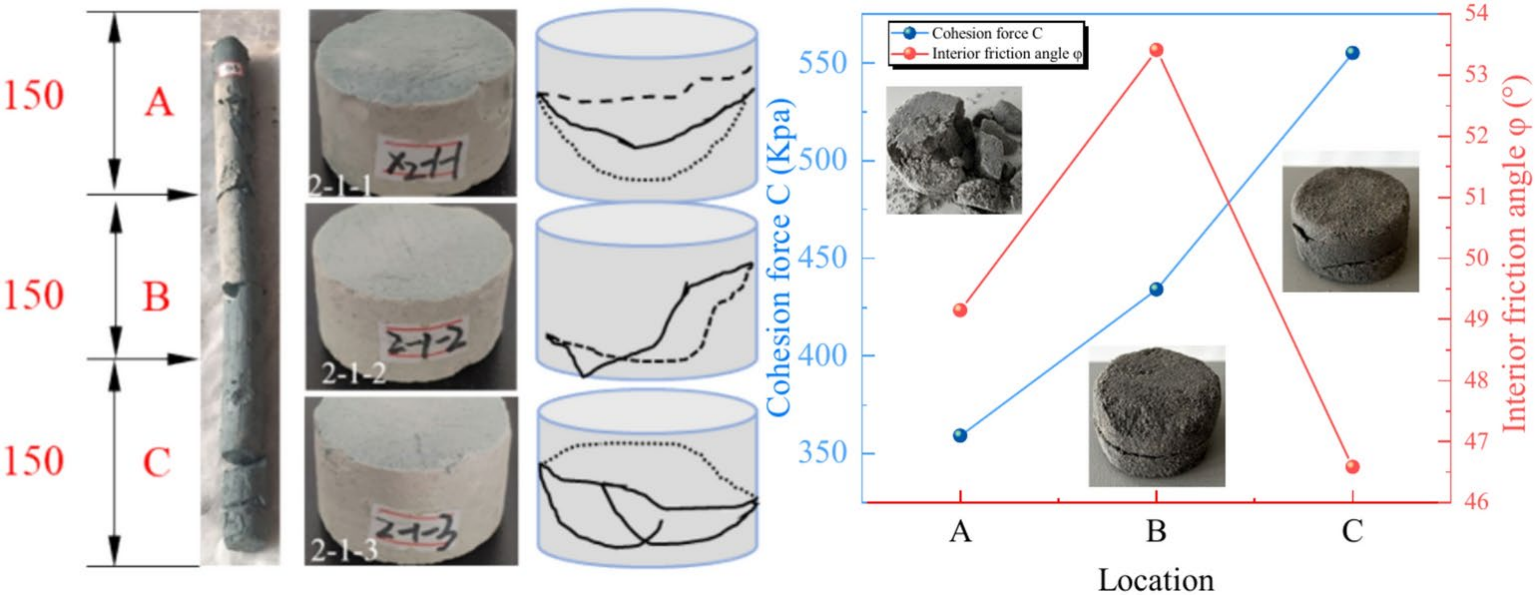
Characteristic diagram of the shear spatial strength: (**a**) failure mode of cracking and (**b**) vertical strength distribution.

For a given section, the shear strength tends to increase with increasing depth. For example, when the depth changes from A to C, the shear strength increases from 1.96 MPa to 9.71 MPa. The images in Fig. 15 clearly show that the generated cracks gradually refine the specimen and yield a porous matrix with depth. Consequently, of the increase in depth played a positive role in the strength of the UCPB.

## Microstructural characteristics of the UCPB

### NMR test of the initial state of the specimen

**Fig.16:**
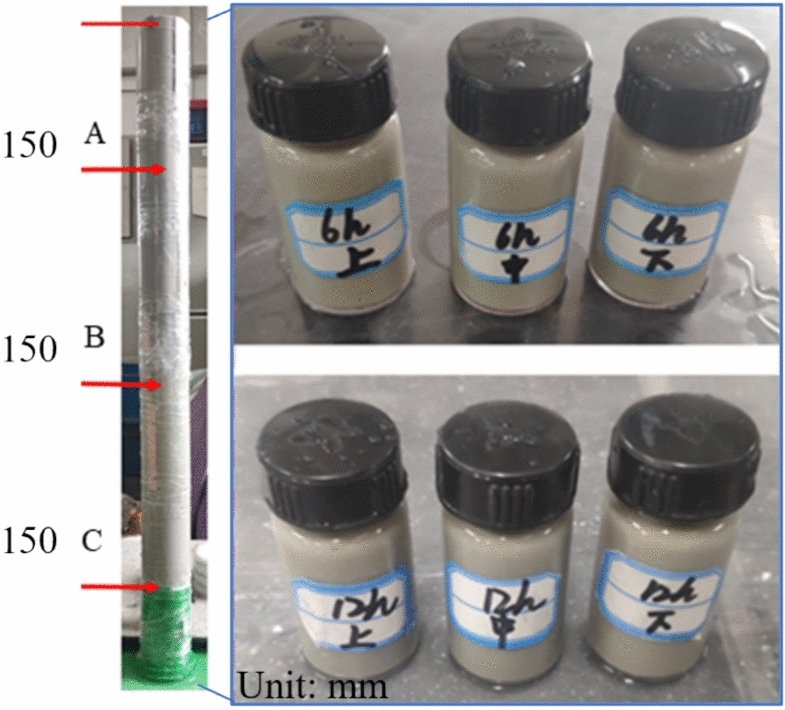
NMR specimens.

**Fig. 17:**
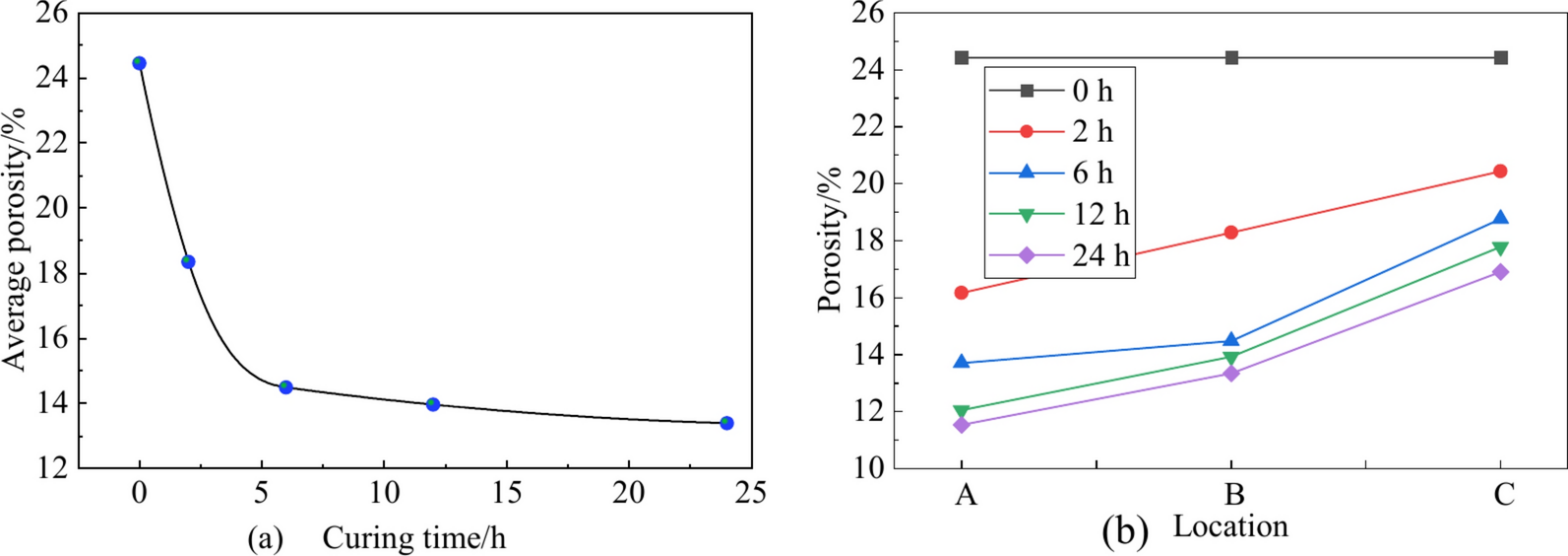
NMR pore tests as a function of (**a**) curing time and (**b**) location.

Figure 16 shows the UCPB slurry of the same material at 0 h, 2 h, 6 h, 12 h, and 24 h maintenance periods, after different amounts were transferred into 30 ml cylindrical glass vials for NMR testing. The precision of the experiment was enhanced by obtaining an average measurement through the creation of three specimens. The results (Fig. 17) show that the main pore size of the UCPB rapidly decreases and then slowly decreases with increasing time. Moreover, the porosity continues to increase with the deepening of the sampling position. The porosity decreased by 45.27% during the entire 24 h of the experiment and by 24.96% during the rapid decline phase. Overall, the porosity of the UCPB decreases with increasing curing time and increases with increasing settlement height.

### CT observation

#### Crack variation pattern

**Fig. 18:**
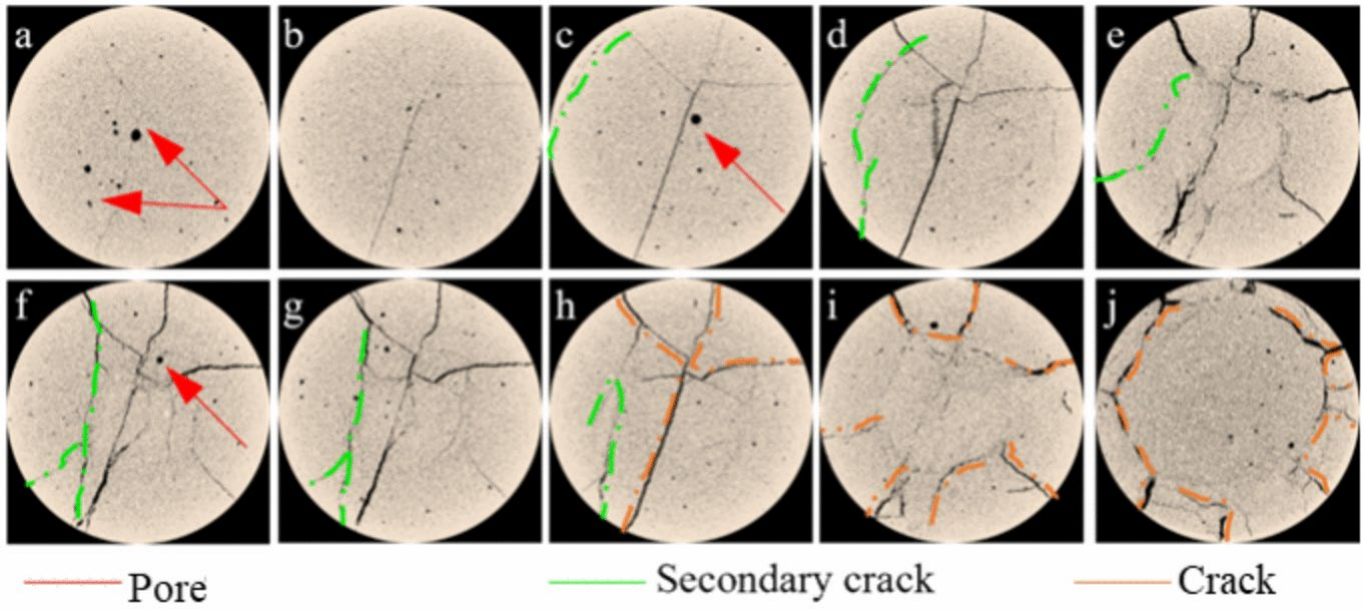
Investigation of X_2_Y_1_ failure modes at various heights from the CT system: (**a**)0 mm ~ (**j**) 100 mm.

To facilitate CT scan observations, we selected specimens filled with material and subjected them to a perimeter pressure of 0.3 MPa. The CT scanning procedure involved capturing a series of cross-sectional slices at 10 mm intervals, spanning from the bottom to the top of the specimens. This comprehensive sequence included sections from the reference surface and the specimen’s base. For analysis, the upper surface was utilized as the reference point. We subsequently processed the acquired scan data via ImageJ software, incorporating image enhancement, filtering, and binarization techniques. This software enabled the meticulous extraction of pores and cracks within the scanned sections, which were subjected to an in-depth analysis. A graphical representation elucidating the distribution of these pores at varying elevations is depicted in Fig. 18.

The image was imported into Avizo, and the 3D Viewer function in the software was used to reconstruct the 3D image. Figure 18 shows the image of each stage in the compression process for 3D reconstruction. The threshold segmentation principle was applied to segment the images of each stage within a specific grayscale range, allowing us to effectively isolate and display the pore structure in the image separately.

Figure 18 also illustrates a “Y-shaped” distribution of cracks, with multiple cracks interconnected by minute pore throats. It is evident that the interconnected pores are well developed in the fractured UCPB, penetrating throughout the entire internal pore structure. Upon examining the sliced images layer by layer Fig. 18(a) ~ (j), it becomes apparent that secondary cracks emerge around primary cracks, isolating the previous "honeycomb-like pore structure." This observation indicates a strong correlation between the vertical dimension size effects and the ultimate failure mode of the UCPB.

On the basis of the damaged structure depicted in Fig. 18, the cracks within the CUPB can be categorized into three types: original pores (red), secondary cracks (green), and primary cracks (orange). The failure mechanism of the UCPB is attributed to stress concentration (as illustrated in Fig. 18, i, and j). Slice studies revealed that no complete through-crack formation occurs under the influence of applied loads at the ends of the specimen. However, a comparison of the slices indicates that these end-acting loads serve as the foundation for developing the main crack, which shares the same direction. Simultaneously, the influence of the original pores is evident in guiding the formation of the main crack structure during the fracture progression, with secondary fractures primarily arising near the main crack. The most distinctive manifestation of the primary and secondary cracks is shown in Fig. 18 (f). The damage evolution manifests as an expansion outwards from the center to the periphery. As the load intensifies, the crack initially arrests and subsequently extends towards the lower boundary, thereby facilitating the dispersion and displacement of UCPB particles.

#### Comparison of porosity

The data obtained through CT scanning display different image pixel grayscale values on the basis of material density. The air (including pores and fractures) was extracted via the threshold segmentation method, and the porosity of the sample was obtained on this basis. The distribution of progressive failure modes in the UCPB under triaxial compression at 0.3 MPa is depicted in Fig. 0.19, which reveals significant variations across distinct regions (Y_1_, Y_3_). In the final phase of the experiment, the test specimen maintains its structural integrity while manifesting localized porosity weakening within its internal framework. The layer-by-layer porosity distribution of the specimen at Y_1_ ranges between 20 and 40%, whereas that at Y_3_ ranges from 0.2% to 0.4%. Tensile failure emerges as the predominant fracture mode in the UCPB test specimen. Notably, there are no discernible voids within the specimen, only dense cracks. This structural configuration imparts greater strength than Y_1_ does.

**Fig. 19:**
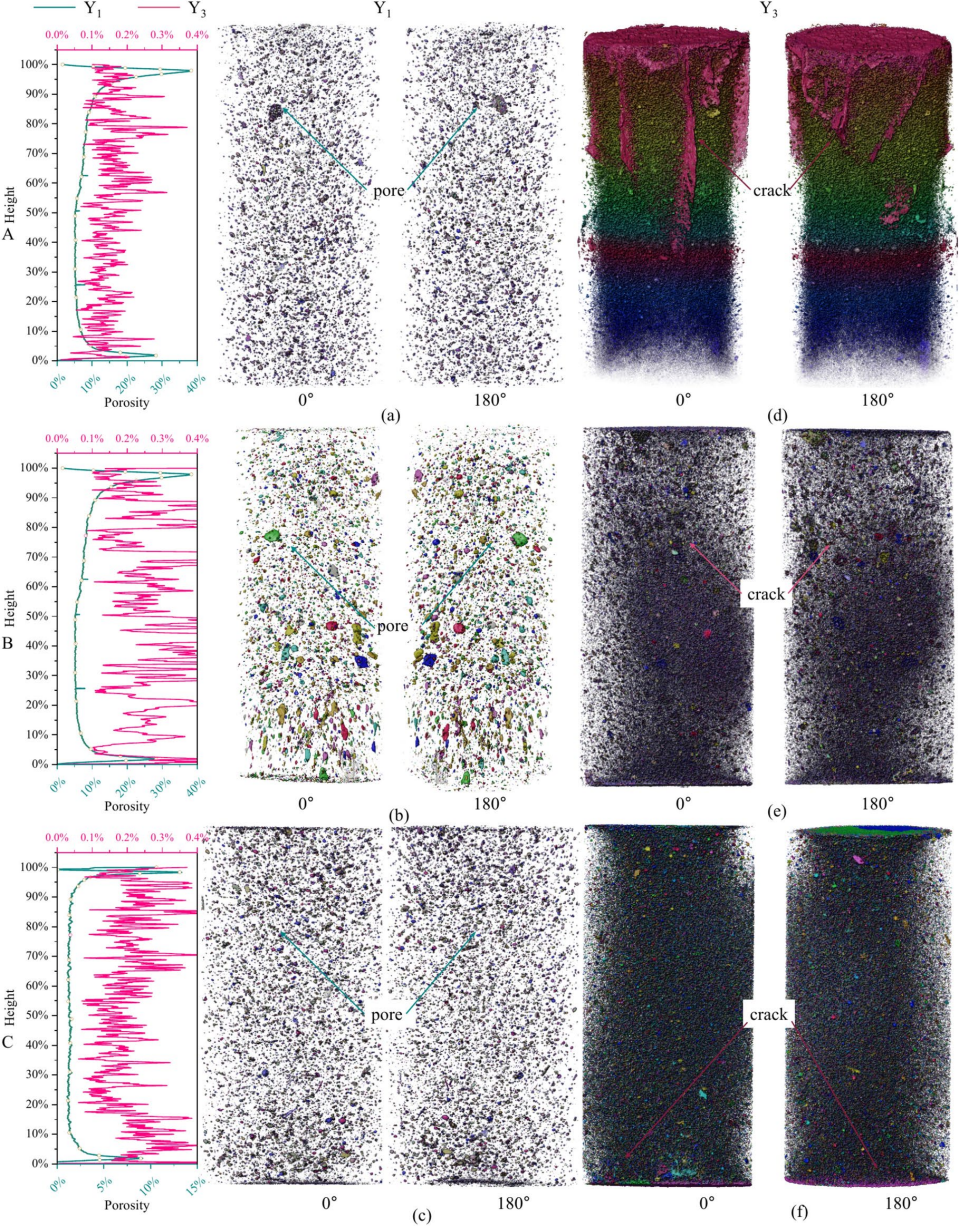
3D reconstruction of the digital images of the specimens at 0.3 MPa for (**a** ~ **c**) Y_1_ and (**e** ~** f**) Y_3_.

As the height of the UCPB decreases, both the surface and side crack diameters gradually increase. Multiple micro-cracks persistently propagate and eventually amplify, culminating in the genesis of through-cracks that precipitate the catastrophic failure of UCPB specimens with specific compositions. By juxtaposing the pore distribution in the same orientation, it becomes apparent that as the depth progresses from A to C, the pore size decreases, concomitant with the porosity decreasing from 10 to 5% (Y_1_) and from 0.4% to 0.3% (Y_3_). Correspondingly, the triaxial strength increases with depth (A-C) and decreases with porosity (Y_1_-Y_3_). Figure 19 suggests that when the UCPB is oriented laterally, microcracks predominantly arise in the axial direction, thereby facilitating accelerated crack propagation. The cracks traverse UCPB, exhibiting extensive crack development, a profusion of deep and broad cracks, with the crack propagation path of the UCPB being limited.

### Particle deposition

#### SEM observation

**Fig. 20:**
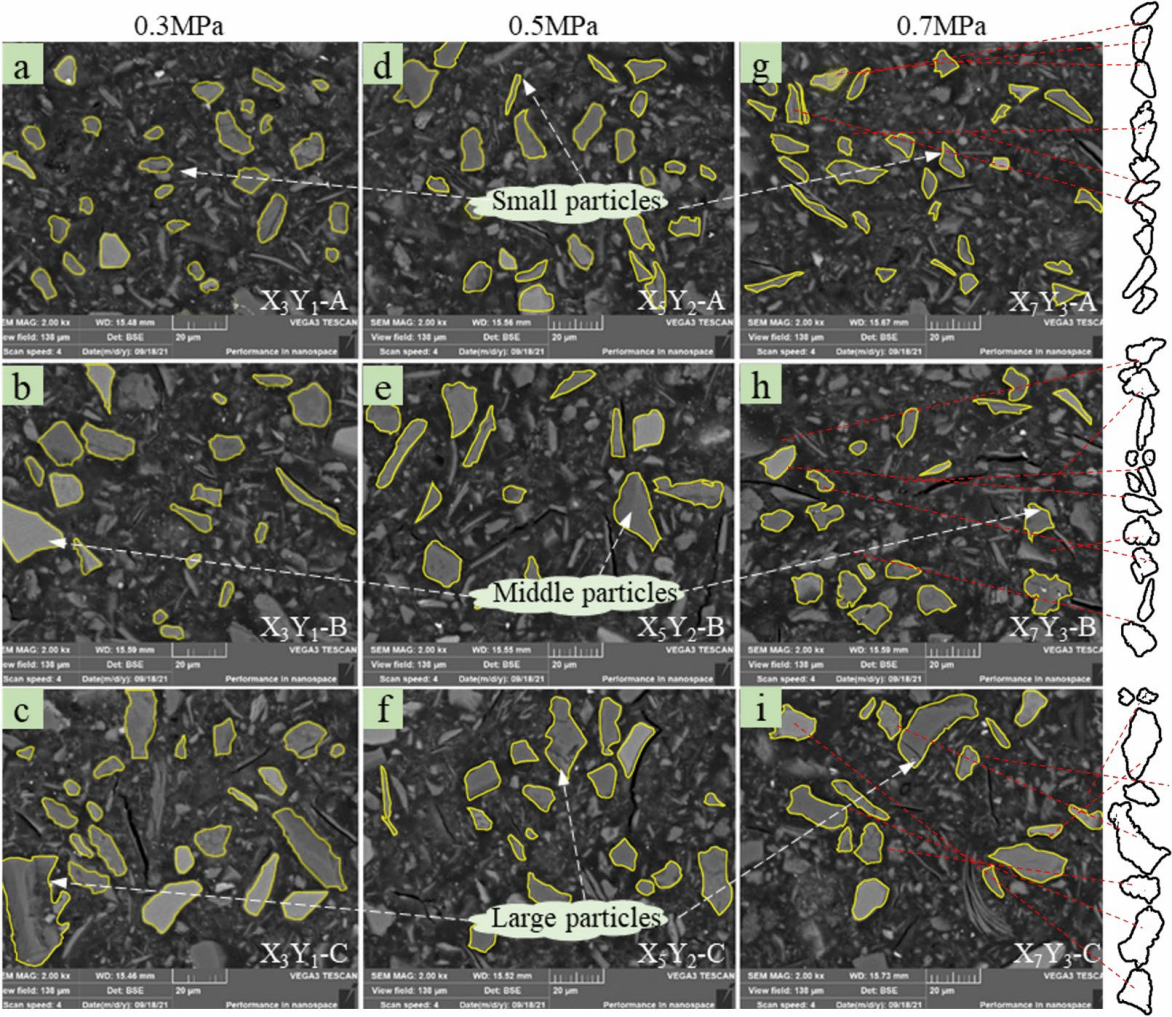
SEM images of broken specimens: (**a**) X_3_Y_1_ ~ (**i**) X_7_Y_3_.

The distribution of fragments further elucidates the effect of the observed size category in polymers on the spatial strength distribution and damage morphological characteristics of the UCPB specimens. The SEM results in Fig. 20 illustrate the distribution of particles after peak loading of three specimens at different heights (X_3_Y_1_, X_5_Y_2_, and X_7_Y_3_). The spatial characteristics of the tailings sand particles are evident, with greater dispersion observed in the upper layers, a significant increase in the number of particles in the middle layer, and the accumulation of larger-sized particles at the bottom. After the destruction of X_7_ was analyzed, a circular area was delineated to represent the analysed fragment particles at various heights. Furthermore, increasing fragmentation of UCPB fossils was observed with increasing depth, indicating that tailings formed occurrence during cement hydration transformation within this interval.

The SEM results revealed that the upper portion depicted was in complete contact with the empty area of Fig. 20. Additionally, a high degree of hydration was exhibited, yielding a backfill material with high strength. Because of the settlement of larger particles, the lower part (C) features smaller pores owing to compaction, resulting in an increase in strength. In conventional triaxial compression tests, the damaged surface tends to flatten as the settlement height decreases.

#### Particle breakage distribution

**Fig. 21:**
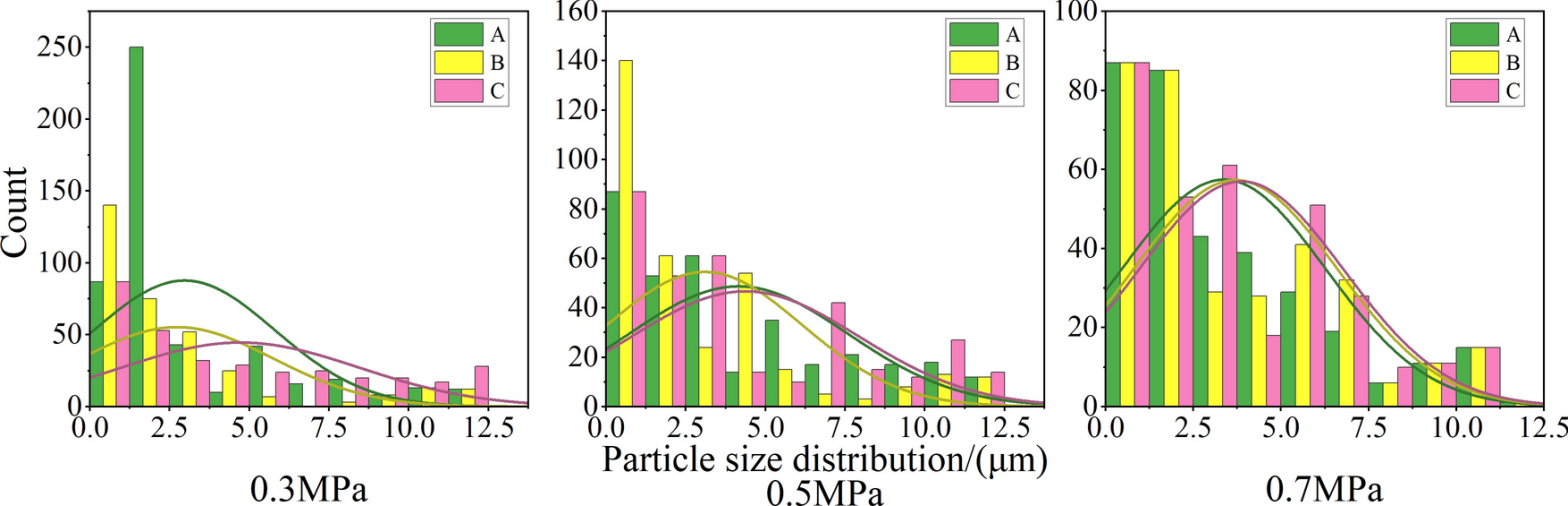
Particle size distributions of the fractured surface.

In X_3_Y_1,_ the average diameter of the sand particles of the upper, middle, and lower tailings was 5.95 μm, as shown in Fig. 21. Similarly, in X_5_Y_2_, the average equivalent diameter of the upper, middle, and lower tailing sand particles was 6.27 μm; in X_7_Y_3_, the average equivalent diameter of the upper, middle, and lower tailing sand particles was 6.27 μm.

Observation of the fragmentation profile of the particles at different heights in drilling X_7_Y_3_ revealed that the particle size gradually increased with decreasing height. The upper portion contained some small-sized particles, with a concentration range between 2 μm and 4 μm, whereas the particles from the central position increased in size, with a concentration range between 4 μm and 6 μm. The bottom portion had a greater proportion of large particles, with a concentration range between 6 μm and 8 μm. The analysis of the curve’s peak points indicates that as the distribution of small particles, which account for the majority of the particle diameter, tends to be neutral, the overall strength of the specimen increases. Conversely, the distribution of large particles in the curve’s tail shows that as the proportion of large particles increases, the particle diameter distribution becomes uneven, resulting in a reduced strength of the filled specimen. The equivalent diameter of drilled particles is negatively correlated with the settling height.

The micropores of the fine particles and the macropores of the coarse particles are the internal pores of the tailings particles and the pores between the tailings and hydration products. Owing to the water absorption of tailings, hydration products can fill the internal pores, and the internal structure of tailings is more compact. On the other hand, they can also be wrapped on the surface of tailings particles to block and fill the pores between particle interfaces, forming a consistent spatial structure to improve compressive strength. However, if the pore size formed by too many large particles is too large, then many hydration products can damage the overall compressive performance. Dense particles with a uniform distribution of hydrated products can coordinate the stress difference of particles under loading. In contrast, the stress difference generated between many hydrated products and large particles under loading cannot be well coordinated and distributed, which is why nonuniform materials are more vulnerable to damage.

## Discussion

Many experts and scholars have studied the spatial distribution law of the strength of the backfill. Xu^40^ studied the non-uniformity of the strength distribution of the backfill with different cement-tailings ratios based on similar simulation experiments. Li^41^ conducted a similar simulation experiment of backfill, and systematically analyzed the distribution law of backfill strength in three different directions. The similar simulation experiment was used to study the strength of backfill, which can effectively verify and observe the mechanical phenomenon of backfill in the stope^42^. Most of the existing studies have studied the distribution law of backfill strength of similar models from one-dimensional or two-dimensional directions. Due to the complexity of the mechanism between the factors affecting the strength of backfill, it is difficult to establish an accurate functional relationship through the regression equation to predict the strength of backfill^43,44^. Some scholars have analyzed the spatial distribution law of particle segregation and backfill strength through neural network^45^. This research method is worth learning and optimizing. Due to the limitation of experimental conditions, we only carry out preliminary research in this field at present. The spatial distribution model of backfill strength and the prediction model of backfill comprehensive strength were not constructed. We will further carry out related research in the future.

Our experiments indicated that a particle size ranging from 6.0 to 6.3 μm resulted in the highest strength UCPB, whereas a tendency towards a medium-sized particle distribution resulted in better performance. The large particles formed the overall structural skeleton inside the UCPB, whereas the small particles filled the pores between the large particles, thereby increasing the compressive strength of the UCPB. Owing to the different particle grades, fine particle micropores and coarse particle macropores can be formed. Small particles with a uniform distribution of hydration products can coordinate the stress difference of the particles under loading, so a uniform particle gradation can render the strength of the UCPB more stable and improve the overall loading performance. However, if too many large particles are formed, the pores between the particles increase, and the hydration products are easy to gather, then the stress difference cannot be well coordinated and distributed between many hydrated products and large particles under loading. It is easy to cause stress concentration, so more large hydration products decrease the overall compressive strength of the material. This is why nonuniform materials are more vulnerable to damage.

The observation results indicate that an increase in the content of fine and coarse aggregates weakens the microstructure of the UCPB specimens, resulting in a more pronounced crack opening due to insufficient support from coarse aggregates. Moreover, weak interfaces between aggregate particles and binding materials with limited hydration products are present. Among all the UCPB specimens, those at the bottom exhibit a superior microstructure characterized by increased hydration products and no apparent defects. The plane area’s central region has the highest microstructural density, indicating its optimal suitability for minimal dissipated energy and the lowest degree of macro-damage after impact.

## Conclusions

In this study, similarity simulation and statistical analysis are utilized to investigate and reveal the spatial distribution of the strength in UCPB under three different stress conditions. This work provides a comprehensive understanding of the evolution of strength and the underlying failure mechanisms in UCPB under various stress scenarios. On the basis of this study, further research can be carried out to build a spatial distribution model of backfill strength and then establish an overall strength prediction model to ensure the strength of the backfill at a construction site. On the basis of the abovementioned findings, we draw the following conclusions:

1. The uniaxial compressive strength of the UCPB averages at 2.22 MPa. In the case of the CTT, the strength increased from 1.35 MPa to 4.69 MPa as the surrounding pressure increased. Among the influencing factors, the vertical distance has the most significant effect on the strength, resulting in higher strength values in the lower region. The strength fluctuates along the material’s flow direction, with the highest values occurring in the middle region. However, the influence of the location’s on strength is insignificant when the direction perpendicular to the flow is considered.
2. Additionally, we observed a distribution pattern of particle sizes within the UCPB specimen; that is, the upper section contained a greater proportion of small-sized particles, the middle section exhibited an increase in particle size with an even distribution, and larger-sized particles were concentrated in the lower section. These findings highlight that the spatial nonuniformity in strength primarily stems from the distribution of hydration products and aggregates within the slurry flow coupled with the particle grading.
3. Our analysis elucidates the mechanism behind spatial distribution and evolutionary patterns by examining microscopic pore characteristics and particle size distributions. Dense particles with a uniform distribution of hydrated products can coordinate the stress difference of particles under loading. In contrast, more large hydration products can make the materials more vulnerable to failure.
4. The test results presented in this study offer a comprehensive database concerning the spatial distribution patterns of strength across various stress conditions and the distribution of grain structures within cemented backfill with ultra-fine tailings. This dataset is valuable for developing and refining physical, mechanical, computational, and numerical models related to CPB.

## Data Availability

All the data generated or analyzed during this study are included in this published article.
